# Bis(2,6-dihy­droxy­benzoato-κ^2^ *O* ^1^ *,O* ^1′^)(nitrato-κ^2^ *O*,*O*′)bis­(1,10-phenanthroline-κ^2^ *N*,*N*′)europium(III)

**DOI:** 10.1107/S1600536810047148

**Published:** 2010-11-20

**Authors:** Bao Huang, Wang Chiya, Wang Xinqing, Hongxiao Jin

**Affiliations:** a College of Materials Science and Engineering, China Jiliang University, Hangzhou 310018, People’s Republic of China

## Abstract

The title mononuclear complex, [Eu(C_7_H_5_O_3_)_2_(NO_3_)(C_12_H_8_N_2_)_2_], is isostructural with those of other lanthanides. The Eu atom is in a pseudo-bicapped square-anti­prismatic geometry, formed by four N atoms from two chelating 1,10-phenanthroline (phen) ligands and by six O atoms, four from two 2,6-dihy­droxy­benzoate (DHB) ligands and the other two from a nitrate anion. π–π stacking inter­actions between phen and DHB ligands [centroid–centroid distances = 3.5312 (19) and 3.8347 (16) Å], and between phen and phen ligands [face-to-face separation = 3.433 (4) Å] of adjacent complexes stabilize the crystal structure. Intra­molecular O—H⋯O hydrogen bonds are observed in the DHB ligands.

## Related literature

For background and details of a related structure, see: Zheng *et al.* (2010 ▶).
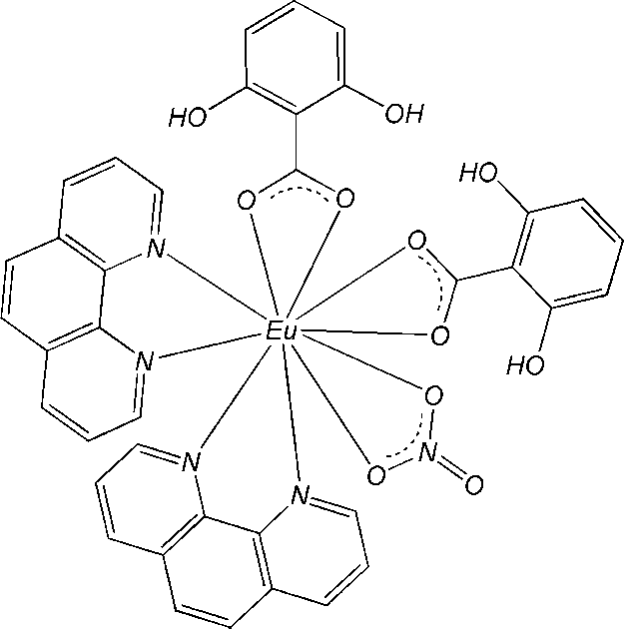


## Experimental

### 

#### Crystal data

[Eu(C_7_H_5_O_3_)_2_(NO_3_)(C_12_H_8_N_2_)_2_]
*M* *_r_* = 880.60
Monoclinic, 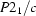

*a* = 11.1855 (2) Å
*b* = 26.7682 (5) Å
*c* = 14.3286 (4) Å
β = 127.557 (2)°
*V* = 3401.05 (16) Å^3^
*Z* = 4
Mo *K*α radiation
μ = 1.92 mm^−1^
*T* = 298 K
0.40 × 0.36 × 0.35 mm

#### Data collection

Oxford Diffraction Gemini S Ultra diffractometer
Absorption correction: multi-scan [*ABSPACK* in *CrysAlis PRO RED* (Oxford Diffraction, 2006 ▶)] *T*_min_ = 0.514, *T*_max_ = 0.553
22346 measured reflections
6936 independent reflections
5281 reflections with *I* > 2σ(*I*)
*R*_int_ = 0.027

#### Refinement

*R*[*F* ^2^ > 2σ(*F* ^2^)] = 0.024
*wR*(*F* ^2^) = 0.043
*S* = 1.05
6936 reflections
496 parameters
8 restraints
H-atom parameters constrained
Δρ_max_ = 0.36 e Å^−3^
Δρ_min_ = −0.49 e Å^−3^

### 

Data collection: *CrysAlis PRO CCD* (Oxford Diffraction, 2006 ▶); cell refinement: *CrysAlis PRO CCD*; data reduction: *CrysAlis PRO RED* (Oxford Diffraction, 2006 ▶); program(s) used to solve structure: *SHELXS97* (Sheldrick, 2008 ▶); program(s) used to refine structure: *SHELXL97* (Sheldrick, 2008 ▶); molecular graphics: *DIAMOND* (Brandenburg & Berndt, 1999 ▶); software used to prepare material for publication: *SHELXL97*.

## Supplementary Material

Crystal structure: contains datablocks I, global. DOI: 10.1107/S1600536810047148/su2227sup1.cif

Structure factors: contains datablocks I. DOI: 10.1107/S1600536810047148/su2227Isup2.hkl

Additional supplementary materials:  crystallographic information; 3D view; checkCIF report

## Figures and Tables

**Table 1:** Hydrogen-bond geometry (Å, °)

*D*—H⋯*A*	*D*—H	H⋯*A*	*D*⋯*A*	*D*—H⋯*A*
O8—H34⋯O6	0.82	1.86	2.585 (3)	148
O4—H27⋯O2	0.82	1.83	2.561 (2)	148
O7—H38⋯O5	0.82	1.85	2.579 (3)	147
O3—H31⋯O1	0.82	1.87	2.594 (2)	147

**Fig. 1.**
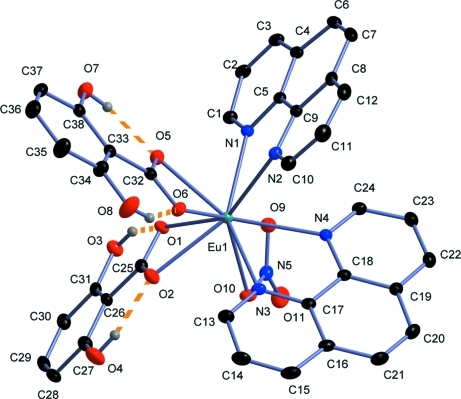
The molecular structure of title compound. Displacement ellipsoids are drawn at the 15% probablility level and H atoms are shown as small spheres of arbitraty radii. Some H atoms are omitted for clarity. Light orange lines show the intramolecular hydrogen bonds (see Table 1 for details).

## supplementary crystallographic information

### Comment

The description of the structure of the title compound is part of a series of papers on mononuclear complexes of the type [Ln(C_12_H_8_N_2_)_2_(C_7_H_8_O_3_)_2_ (NO_3_)], with Ln = Ce, Pr, Sm, Eu (this publication), and Dy. All five compounds are isostructural to the previously reported Nd complex (Zheng *et al.* 2010). The background to this study is given in the previous paper by Zheng *et al.* (2010).

### Experimental

Each reagent was commercially available and of analytical grade. Eu(NO_3_)_3_^.^6H_2_O (0.224 g, 0.5 mmol), 2, 6-dihydroxybenzoic acid (0.074 g 0.5 mmol), 1, 10-phenanthroline (0.090 g, 0.5 mmol) and NaHCO_3_ (0.042 g, 0.5 mmol) were dissolved in water-ethanol solution (10 ml, 5:5). The solution was refluxed for 4 h, and filtered after cooling to room temperature. Orange single crystals were obtained from the filtrate after 3 d.

### Refinement

H atoms were positioned geometrically (C—H = 0.93 Å and O—H = 0.82 Å) and refined as riding, with *U*_iso _(H) = 1.2Ueq (C) and *U*_iso_(H) = 1.5Ueq (O). The anisotropic displacement of the O10, N5, Eu1 and O9 atoms were restrained to be equal.

### Crystal data

**Table:**

[Eu(C_7_H_5_O_3_)_2_(NO_3_)(C_12_H_8_N_2_)_2_]	*F*(000) = 1760
*M**_r_* = 880.60	*D*_x_ = 1.720 Mg m^−^^3^
Monoclinic, *P*2_1_/*c*	Mo *K*α radiation, λ = 0.71073 Å
Hall symbol: -P 2ybc	Cell parameters from 11999 reflections
*a* = 11.1855 (2) Å	θ = 2.9–29.1°
*b* = 26.7682 (5) Å	µ = 1.92 mm^−^^1^
*c* = 14.3286 (4) Å	*T* = 298 K
β = 127.557 (2)°	Block, orange
*V* = 3401.05 (16) Å^3^	0.40 × 0.36 × 0.35 mm
*Z* = 4	

### Data collection

**Table:**

Oxford Diffraction Gemini S Ultra diffractometer	6936 independent reflections
Radiation source: Enhance (Mo) X-ray Source	5281 reflections with *I* > 2σ(*I*)
graphite	*R*_int_ = 0.027
Detector resolution: 15.9149 pixels mm^-1^	θ_max_ = 26.4°, θ_min_ = 3.0°
ω scans	*h* = −13→10
Absorption correction: multi-scan [*ABSPACK* in *CrysAlis PRO RED* (Oxford Diffraction, 2006)]	*k* = −33→32
*T*_min_ = 0.514, *T*_max_ = 0.553	*l* = −11→17
22346 measured reflections	

### Refinement

**Table:**

Refinement on *F*^2^	Primary atom site location: structure-invariant direct methods
Least-squares matrix: full	Secondary atom site location: difference Fourier map
*R*[*F*^2^ > 2σ(*F*^2^)] = 0.024	Hydrogen site location: inferred from neighbouring sites
*wR*(*F*^2^) = 0.043	H-atom parameters constrained
*S* = 1.05	*w* = 1/[σ^2^(*F*_o_^2^) + (0.0144*P*)^2^] where *P* = (*F*_o_^2^ + 2*F*_c_^2^)/3
6936 reflections	(Δ/σ)_max_ = 0.001
496 parameters	Δρ_max_ = 0.36 e Å^−^^3^
8 restraints	Δρ_min_ = −0.49 e Å^−^^3^

### Special details

**Table:**

Geometry. All e.s.d.'s (except the e.s.d. in the dihedral angle between two l.s. planes) are estimated using the full covariance matrix. The cell e.s.d.'s are taken into account individually in the estimation of e.s.d.'s in distances, angles and torsion angles; correlations between e.s.d.'s in cell parameters are only used when they are defined by crystal symmetry. An approximate (isotropic) treatment of cell e.s.d.'s is used for estimating e.s.d.'s involving l.s. planes.
Refinement. Refinement of *F*^2^ against ALL reflections. The weighted *R*-factor *wR* and goodness of fit *S* are based on *F*^2^, conventional *R*-factors *R* are based on *F*, with *F* set to zero for negative *F*^2^. The threshold expression of *F*^2^ > σ(*F*^2^) is used only for calculating *R*-factors(gt) *etc*. and is not relevant to the choice of reflections for refinement. *R*-factors based on *F*^2^ are statistically about twice as large as those based on *F*, and *R*- factors based on ALL data will be even larger.

### Fractional atomic coordinates and isotropic or equivalent isotropic displacement parameters (Å^2^)

**Table:**

	*x*	*y*	*z*	*U*_iso_*/*U*_eq_	
Eu1	0.430778 (13)	0.861975 (4)	0.720853 (12)	0.03240 (4)	
O1	0.19590 (17)	0.81211 (6)	0.63374 (15)	0.0437 (5)	
O2	0.16583 (18)	0.89279 (7)	0.59806 (16)	0.0492 (5)	
O3	−0.01921 (19)	0.75869 (7)	0.60271 (16)	0.0535 (5)	
H31	0.0624	0.7641	0.6166	0.080*	
O4	−0.0800 (2)	0.93598 (7)	0.5290 (2)	0.0750 (7)	
H27	0.0062	0.9333	0.5499	0.113*	
O5	0.34598 (18)	0.83820 (7)	0.52114 (15)	0.0447 (5)	
O6	0.38918 (18)	0.91856 (6)	0.55787 (15)	0.0448 (5)	
O7	0.2549 (2)	0.80247 (7)	0.32044 (17)	0.0606 (5)	
H38	0.2789	0.8021	0.3871	0.091*	
O8	0.3356 (3)	0.97889 (7)	0.39552 (19)	0.0816 (7)	
H34	0.3566	0.9705	0.4590	0.122*	
O9	0.45362 (19)	0.80112 (6)	0.87237 (16)	0.0469 (4)	
O10	0.34644 (19)	0.87175 (7)	0.84764 (17)	0.0503 (5)	
O11	0.3634 (2)	0.81721 (9)	0.96677 (19)	0.0823 (7)	
N1	0.5570 (2)	0.77913 (7)	0.73843 (17)	0.0347 (5)	
N2	0.6809 (2)	0.86747 (7)	0.74804 (18)	0.0370 (5)	
N3	0.4755 (2)	0.95459 (7)	0.79134 (19)	0.0370 (5)	
N4	0.6651 (2)	0.88072 (7)	0.93947 (18)	0.0355 (5)	
N5	0.3879 (2)	0.82911 (9)	0.8983 (2)	0.0463 (5)	
C1	0.4920 (3)	0.73535 (10)	0.7235 (2)	0.0427 (7)	
H1	0.3946	0.7352	0.7013	0.051*	
C2	0.5611 (3)	0.68954 (10)	0.7391 (2)	0.0478 (7)	
H2	0.5095	0.6598	0.7246	0.057*	
C3	0.7046 (3)	0.68922 (10)	0.7756 (2)	0.0480 (7)	
H3	0.7532	0.6590	0.7877	0.058*	
C4	0.7801 (3)	0.73409 (10)	0.7951 (2)	0.0417 (7)	
C5	0.7001 (3)	0.77846 (9)	0.7716 (2)	0.0347 (6)	
C6	0.9345 (3)	0.73719 (12)	0.8396 (2)	0.0526 (8)	
H6	0.9904	0.7080	0.8605	0.063*	
C7	1.0004 (3)	0.78094 (12)	0.8518 (2)	0.0538 (8)	
H7	1.1009	0.7816	0.8812	0.065*	
C8	0.9179 (3)	0.82669 (11)	0.8201 (2)	0.0437 (7)	
C9	0.7677 (3)	0.82588 (10)	0.7813 (2)	0.0360 (6)	
C10	0.7415 (3)	0.91061 (10)	0.7505 (2)	0.0470 (7)	
H10	0.6829	0.9393	0.7266	0.056*	
C11	0.8890 (3)	0.91501 (12)	0.7871 (3)	0.0552 (8)	
H11	0.9270	0.9459	0.7871	0.066*	
C12	0.9755 (3)	0.87350 (12)	0.8224 (3)	0.0549 (8)	
H12	1.0744	0.8761	0.8486	0.066*	
C13	0.3819 (3)	0.99087 (10)	0.7204 (3)	0.0465 (7)	
H13	0.3059	0.9833	0.6418	0.056*	
C14	0.3929 (3)	1.03979 (10)	0.7587 (3)	0.0532 (8)	
H14	0.3254	1.0640	0.7062	0.064*	
C15	0.5022 (3)	1.05169 (10)	0.8724 (3)	0.0520 (8)	
H15	0.5099	1.0842	0.8985	0.062*	
C16	0.6039 (3)	1.01513 (9)	0.9512 (3)	0.0406 (7)	
C17	0.5851 (3)	0.96642 (9)	0.9062 (2)	0.0341 (6)	
C18	0.6860 (3)	0.92762 (9)	0.9847 (2)	0.0337 (6)	
C19	0.8009 (3)	0.93855 (10)	1.1035 (2)	0.0393 (6)	
C20	0.8156 (3)	0.98871 (11)	1.1446 (3)	0.0507 (8)	
H20	0.8923	0.9962	1.2231	0.061*	
C21	0.7213 (3)	1.02491 (11)	1.0723 (3)	0.0509 (8)	
H21	0.7326	1.0570	1.1017	0.061*	
C22	0.8964 (3)	0.89993 (11)	1.1759 (3)	0.0501 (8)	
H22	0.9733	0.9060	1.2552	0.060*	
C23	0.8775 (3)	0.85332 (11)	1.1308 (2)	0.0477 (7)	
H23	0.9415	0.8273	1.1780	0.057*	
C24	0.7604 (3)	0.84565 (10)	1.0125 (2)	0.0418 (7)	
H24	0.7479	0.8137	0.9825	0.050*	
C25	0.1141 (3)	0.85088 (10)	0.5999 (2)	0.0405 (7)	
C26	−0.0402 (3)	0.84766 (10)	0.5660 (2)	0.0388 (7)	
C27	−0.1308 (3)	0.89040 (12)	0.5326 (3)	0.0521 (8)	
C28	−0.2734 (3)	0.88738 (13)	0.5031 (3)	0.0586 (8)	
H28	−0.3331	0.9158	0.4806	0.070*	
C29	−0.3250 (3)	0.84196 (14)	0.5077 (3)	0.0586 (9)	
H29	−0.4210	0.8400	0.4878	0.070*	
C30	−0.2418 (3)	0.79931 (12)	0.5402 (2)	0.0516 (8)	
H30	−0.2807	0.7690	0.5422	0.062*	
C31	−0.0983 (3)	0.80167 (11)	0.5701 (2)	0.0414 (7)	
C32	0.3466 (3)	0.88224 (11)	0.4875 (2)	0.0368 (6)	
C33	0.2975 (2)	0.89004 (10)	0.3666 (2)	0.0361 (6)	
C34	0.2936 (3)	0.93810 (11)	0.3255 (3)	0.0499 (7)	
C35	0.2441 (3)	0.94544 (12)	0.2110 (3)	0.0641 (9)	
H35	0.2395	0.9775	0.1840	0.077*	
C36	0.2023 (3)	0.90549 (13)	0.1386 (3)	0.0609 (8)	
H36	0.1703	0.9107	0.0623	0.073*	
C37	0.2059 (3)	0.85796 (12)	0.1748 (2)	0.0520 (7)	
H37	0.1767	0.8313	0.1235	0.062*	
C38	0.2537 (3)	0.84956 (10)	0.2888 (2)	0.0409 (7)	

### Atomic displacement parameters (Å^2^)

**Table:**

	*U*^11^	*U*^22^	*U*^33^	*U*^12^	*U*^13^	*U*^23^
Eu1	0.03389 (7)	0.02771 (7)	0.03845 (8)	0.00054 (6)	0.02352 (6)	−0.00013 (7)
O1	0.0357 (9)	0.0386 (11)	0.0556 (12)	0.0033 (8)	0.0273 (10)	0.0001 (9)
O2	0.0405 (10)	0.0420 (12)	0.0621 (13)	0.0030 (9)	0.0297 (10)	0.0085 (10)
O3	0.0439 (11)	0.0508 (13)	0.0675 (14)	0.0003 (9)	0.0348 (11)	0.0069 (11)
O4	0.0591 (13)	0.0547 (14)	0.1005 (19)	0.0183 (11)	0.0431 (13)	0.0140 (13)
O5	0.0501 (11)	0.0397 (11)	0.0416 (12)	−0.0016 (9)	0.0265 (10)	0.0019 (10)
O6	0.0564 (11)	0.0396 (11)	0.0391 (11)	−0.0023 (9)	0.0296 (10)	−0.0027 (9)
O7	0.0716 (13)	0.0416 (12)	0.0486 (13)	−0.0009 (10)	0.0262 (11)	−0.0062 (10)
O8	0.140 (2)	0.0422 (13)	0.0585 (15)	−0.0165 (13)	0.0586 (16)	−0.0025 (12)
O9	0.0533 (11)	0.0388 (10)	0.0519 (12)	0.0002 (8)	0.0337 (10)	0.0046 (8)
O10	0.0594 (11)	0.0476 (10)	0.0605 (13)	0.0028 (8)	0.0451 (11)	−0.0043 (9)
O11	0.0823 (15)	0.128 (2)	0.0583 (14)	−0.0063 (14)	0.0539 (14)	0.0139 (15)
N1	0.0360 (12)	0.0318 (12)	0.0384 (13)	0.0002 (9)	0.0237 (11)	−0.0006 (10)
N2	0.0396 (11)	0.0371 (13)	0.0393 (13)	−0.0055 (10)	0.0266 (11)	−0.0033 (11)
N3	0.0479 (13)	0.0266 (12)	0.0450 (14)	0.0023 (10)	0.0327 (13)	0.0031 (10)
N4	0.0355 (11)	0.0332 (12)	0.0380 (13)	0.0019 (9)	0.0226 (11)	−0.0005 (10)
N5	0.0444 (13)	0.0586 (13)	0.0406 (14)	−0.0066 (10)	0.0283 (12)	−0.0014 (10)
C1	0.0478 (15)	0.0354 (17)	0.0466 (18)	−0.0015 (13)	0.0296 (15)	−0.0042 (13)
C2	0.0647 (19)	0.0301 (16)	0.0493 (18)	0.0017 (13)	0.0350 (17)	−0.0018 (13)
C3	0.0651 (19)	0.0364 (17)	0.0438 (18)	0.0136 (14)	0.0340 (17)	0.0025 (14)
C4	0.0456 (16)	0.0502 (18)	0.0327 (16)	0.0122 (13)	0.0256 (15)	0.0043 (13)
C5	0.0372 (14)	0.0394 (16)	0.0321 (15)	0.0023 (11)	0.0235 (13)	−0.0010 (12)
C6	0.0502 (18)	0.063 (2)	0.0441 (19)	0.0221 (15)	0.0284 (16)	0.0057 (16)
C7	0.0343 (15)	0.085 (2)	0.0408 (18)	0.0103 (16)	0.0225 (15)	0.0000 (17)
C8	0.0352 (15)	0.064 (2)	0.0330 (16)	−0.0059 (14)	0.0212 (14)	−0.0075 (15)
C9	0.0338 (14)	0.0464 (17)	0.0315 (15)	−0.0006 (12)	0.0217 (13)	−0.0023 (13)
C10	0.0507 (17)	0.0450 (17)	0.0501 (18)	−0.0075 (13)	0.0331 (15)	−0.0038 (15)
C11	0.0535 (18)	0.057 (2)	0.063 (2)	−0.0255 (15)	0.0395 (18)	−0.0108 (17)
C12	0.0379 (16)	0.083 (3)	0.0469 (18)	−0.0156 (16)	0.0276 (15)	−0.0108 (17)
C13	0.0572 (17)	0.0358 (17)	0.0544 (19)	0.0067 (13)	0.0381 (16)	0.0061 (14)
C14	0.072 (2)	0.0307 (17)	0.077 (2)	0.0110 (14)	0.055 (2)	0.0121 (16)
C15	0.078 (2)	0.0277 (16)	0.083 (2)	−0.0057 (15)	0.065 (2)	−0.0058 (16)
C16	0.0485 (16)	0.0307 (15)	0.063 (2)	−0.0079 (12)	0.0446 (17)	−0.0059 (14)
C17	0.0390 (14)	0.0311 (15)	0.0476 (18)	−0.0050 (11)	0.0343 (15)	−0.0015 (13)
C18	0.0353 (14)	0.0344 (15)	0.0443 (17)	−0.0052 (11)	0.0309 (14)	−0.0043 (13)
C19	0.0358 (14)	0.0451 (17)	0.0438 (17)	−0.0078 (12)	0.0278 (15)	−0.0076 (14)
C20	0.0481 (17)	0.058 (2)	0.0510 (19)	−0.0212 (15)	0.0329 (16)	−0.0210 (17)
C21	0.0608 (19)	0.0393 (18)	0.073 (2)	−0.0197 (15)	0.051 (2)	−0.0208 (17)
C22	0.0344 (15)	0.067 (2)	0.0441 (18)	−0.0054 (14)	0.0215 (15)	−0.0055 (16)
C23	0.0399 (15)	0.055 (2)	0.0453 (18)	0.0058 (13)	0.0246 (15)	0.0067 (15)
C24	0.0468 (16)	0.0373 (16)	0.0457 (18)	0.0033 (12)	0.0305 (16)	0.0011 (13)
C25	0.0392 (15)	0.0458 (19)	0.0357 (16)	0.0008 (12)	0.0225 (14)	0.0008 (13)
C26	0.0311 (14)	0.0479 (18)	0.0332 (15)	0.0050 (11)	0.0173 (13)	0.0018 (12)
C27	0.0479 (17)	0.056 (2)	0.0490 (19)	0.0092 (15)	0.0276 (16)	0.0075 (16)
C28	0.0417 (17)	0.079 (2)	0.052 (2)	0.0234 (16)	0.0270 (16)	0.0071 (18)
C29	0.0315 (15)	0.100 (3)	0.0434 (19)	0.0068 (17)	0.0222 (15)	0.0006 (18)
C30	0.0381 (16)	0.077 (2)	0.0431 (18)	−0.0037 (15)	0.0263 (15)	0.0016 (16)
C31	0.0345 (14)	0.0577 (19)	0.0290 (15)	0.0024 (13)	0.0178 (13)	0.0011 (14)
C32	0.0294 (13)	0.0420 (16)	0.0394 (17)	0.0022 (12)	0.0212 (13)	0.0022 (14)
C33	0.0283 (13)	0.0411 (16)	0.0367 (16)	0.0014 (11)	0.0188 (13)	0.0018 (13)
C34	0.0619 (18)	0.0447 (19)	0.0458 (19)	−0.0078 (14)	0.0342 (17)	−0.0021 (15)
C35	0.087 (2)	0.058 (2)	0.051 (2)	−0.0151 (18)	0.044 (2)	0.0030 (18)
C36	0.066 (2)	0.076 (2)	0.0427 (19)	−0.0080 (17)	0.0346 (17)	0.0005 (18)
C37	0.0486 (16)	0.063 (2)	0.0440 (18)	−0.0028 (15)	0.0280 (15)	−0.0130 (17)
C38	0.0293 (13)	0.0459 (19)	0.0410 (17)	0.0016 (11)	0.0182 (14)	−0.0014 (13)

### Geometric parameters (Å, °)

**Table:**

Eu1—O5	2.4869 (17)	C8—C12	1.400 (4)
Eu1—O2	2.4904 (16)	C8—C9	1.413 (3)
Eu1—O1	2.4989 (16)	C10—C11	1.399 (3)
Eu1—O10	2.5196 (17)	C10—H10	0.9300
Eu1—N1	2.5574 (19)	C11—C12	1.352 (4)
Eu1—O6	2.5767 (17)	C11—H11	0.9300
Eu1—N2	2.5830 (18)	C12—H12	0.9300
Eu1—O9	2.5991 (17)	C13—C14	1.396 (3)
Eu1—N3	2.608 (2)	C13—H13	0.9300
Eu1—N4	2.632 (2)	C14—C15	1.350 (4)
Eu1—C25	2.856 (3)	C14—H14	0.9300
Eu1—C32	2.919 (3)	C15—C16	1.400 (4)
O1—C25	1.269 (3)	C15—H15	0.9300
O2—C25	1.270 (3)	C16—C17	1.412 (3)
O3—C31	1.349 (3)	C16—C21	1.424 (4)
O3—H31	0.8199	C17—C18	1.439 (3)
O4—C27	1.361 (3)	C18—C19	1.403 (3)
O4—H27	0.8200	C19—C22	1.391 (3)
O5—C32	1.275 (3)	C19—C20	1.435 (4)
O6—C32	1.266 (3)	C20—C21	1.339 (4)
O7—C38	1.337 (3)	C20—H20	0.9300
O7—H38	0.8200	C21—H21	0.9300
O8—C34	1.358 (3)	C22—C23	1.361 (4)
O8—H34	0.8200	C22—H22	0.9300
O9—N5	1.254 (3)	C23—C24	1.387 (4)
O10—N5	1.278 (3)	C23—H23	0.9300
O11—N5	1.211 (3)	C24—H24	0.9300
N1—C1	1.326 (3)	C25—C26	1.482 (3)
N1—C5	1.365 (3)	C26—C27	1.405 (3)
N2—C10	1.329 (3)	C26—C31	1.411 (3)
N2—C9	1.358 (3)	C27—C28	1.381 (3)
N3—C13	1.332 (3)	C28—C29	1.365 (4)
N3—C17	1.361 (3)	C28—H28	0.9300
N4—C24	1.324 (3)	C29—C30	1.363 (4)
N4—C18	1.366 (3)	C29—H29	0.9300
C1—C2	1.393 (3)	C30—C31	1.387 (3)
C1—H1	0.9300	C30—H30	0.9300
C2—C3	1.352 (3)	C32—C33	1.479 (3)
C2—H2	0.9300	C33—C34	1.405 (3)
C3—C4	1.395 (4)	C33—C38	1.411 (3)
C3—H3	0.9300	C34—C35	1.390 (4)
C4—C5	1.399 (3)	C35—C36	1.359 (4)
C4—C6	1.433 (3)	C35—H35	0.9300
C5—C9	1.440 (3)	C36—C37	1.365 (4)
C6—C7	1.337 (4)	C36—H36	0.9300
C6—H6	0.9300	C37—C38	1.394 (4)
C7—C8	1.430 (4)	C37—H37	0.9300
C7—H7	0.9300		
			
O5—Eu1—O2	79.45 (6)	N1—C5—C9	117.2 (2)
O5—Eu1—O1	74.99 (6)	C4—C5—C9	120.3 (2)
O2—Eu1—O1	52.27 (6)	C7—C6—C4	121.8 (3)
O5—Eu1—O10	143.94 (6)	C7—C6—H6	119.1
O2—Eu1—O10	70.59 (6)	C4—C6—H6	119.1
O1—Eu1—O10	70.97 (6)	C6—C7—C8	120.7 (2)
O5—Eu1—N1	72.05 (6)	C6—C7—H7	119.6
O2—Eu1—N1	134.78 (6)	C8—C7—H7	119.6
O1—Eu1—N1	86.28 (6)	C12—C8—C9	116.3 (3)
O10—Eu1—N1	116.47 (6)	C12—C8—C7	124.1 (2)
O5—Eu1—O6	51.40 (6)	C9—C8—C7	119.6 (2)
O2—Eu1—O6	71.65 (5)	N2—C9—C8	123.3 (2)
O1—Eu1—O6	107.90 (6)	N2—C9—C5	117.9 (2)
O10—Eu1—O6	130.72 (6)	C8—C9—C5	118.7 (2)
N1—Eu1—O6	112.52 (6)	N2—C10—C11	123.3 (3)
O5—Eu1—N2	78.84 (6)	N2—C10—H10	118.3
O2—Eu1—N2	143.20 (6)	C11—C10—H10	118.3
O1—Eu1—N2	144.89 (6)	C12—C11—C10	118.8 (3)
O10—Eu1—N2	137.10 (6)	C12—C11—H11	120.6
N1—Eu1—N2	63.42 (6)	C10—C11—H11	120.6
O6—Eu1—N2	71.57 (6)	C11—C12—C8	120.9 (2)
O5—Eu1—O9	125.11 (6)	C11—C12—H12	119.6
O2—Eu1—O9	105.46 (6)	C8—C12—H12	119.6
O1—Eu1—O9	67.70 (6)	N3—C13—C14	122.9 (3)
O10—Eu1—O9	49.62 (6)	N3—C13—H13	118.5
N1—Eu1—O9	66.85 (6)	C14—C13—H13	118.5
O6—Eu1—O9	175.49 (5)	C15—C14—C13	119.6 (3)
N2—Eu1—O9	111.33 (6)	C15—C14—H14	120.2
O5—Eu1—N3	122.52 (6)	C13—C14—H14	120.2
O2—Eu1—N3	79.83 (6)	C14—C15—C16	120.1 (3)
O1—Eu1—N3	126.60 (6)	C14—C15—H15	120.0
O10—Eu1—N3	71.92 (6)	C16—C15—H15	120.0
N1—Eu1—N3	145.31 (6)	C15—C16—C17	117.1 (3)
O6—Eu1—N3	71.23 (6)	C15—C16—C21	123.1 (3)
N2—Eu1—N3	87.31 (6)	C17—C16—C21	119.8 (3)
O9—Eu1—N3	111.98 (6)	N3—C17—C16	122.7 (2)
O5—Eu1—N4	145.39 (6)	N3—C17—C18	118.5 (2)
O2—Eu1—N4	132.12 (6)	C16—C17—C18	118.8 (2)
O1—Eu1—N4	132.65 (6)	N4—C18—C19	122.2 (2)
O10—Eu1—N4	70.18 (6)	N4—C18—C17	117.8 (2)
N1—Eu1—N4	87.63 (6)	C19—C18—C17	120.0 (2)
O6—Eu1—N4	117.73 (6)	C22—C19—C18	118.0 (2)
N2—Eu1—N4	66.94 (6)	C22—C19—C20	123.1 (3)
O9—Eu1—N4	66.78 (6)	C18—C19—C20	119.0 (3)
N3—Eu1—N4	62.68 (6)	C21—C20—C19	121.4 (3)
O5—Eu1—C25	79.24 (6)	C21—C20—H20	119.3
O2—Eu1—C25	26.36 (6)	C19—C20—H20	119.3
O1—Eu1—C25	26.34 (6)	C20—C21—C16	121.1 (3)
O10—Eu1—C25	64.98 (7)	C20—C21—H21	119.4
N1—Eu1—C25	112.06 (7)	C16—C21—H21	119.4
O6—Eu1—C25	92.25 (6)	C23—C22—C19	120.1 (3)
N2—Eu1—C25	157.87 (7)	C23—C22—H22	120.0
O9—Eu1—C25	84.01 (7)	C19—C22—H22	120.0
N3—Eu1—C25	102.00 (7)	C22—C23—C24	118.2 (3)
N4—Eu1—C25	135.16 (7)	C22—C23—H23	120.9
O5—Eu1—C32	25.73 (6)	C24—C23—H23	120.9
O2—Eu1—C32	73.44 (6)	N4—C24—C23	124.5 (2)
O1—Eu1—C32	91.15 (6)	N4—C24—H24	117.7
O10—Eu1—C32	143.64 (6)	C23—C24—H24	117.7
N1—Eu1—C32	92.71 (7)	O1—C25—O2	119.9 (2)
O6—Eu1—C32	25.68 (6)	O1—C25—C26	120.1 (2)
N2—Eu1—C32	74.09 (6)	O2—C25—C26	119.9 (2)
O9—Eu1—C32	150.69 (7)	O1—C25—Eu1	60.92 (12)
N3—Eu1—C32	96.82 (7)	O2—C25—Eu1	60.54 (12)
N4—Eu1—C32	136.05 (6)	C26—C25—Eu1	165.99 (18)
C25—Eu1—C32	84.81 (7)	C27—C26—C31	118.0 (2)
C25—O1—Eu1	92.74 (14)	C27—C26—C25	121.2 (2)
C25—O2—Eu1	93.09 (15)	C31—C26—C25	120.8 (2)
C31—O3—H31	109.5	O4—C27—C28	118.2 (3)
C27—O4—H27	109.4	O4—C27—C26	120.7 (2)
C32—O5—Eu1	96.44 (16)	C28—C27—C26	121.1 (3)
C32—O6—Eu1	92.45 (15)	C29—C28—C27	118.7 (3)
C38—O7—H38	109.5	C29—C28—H28	120.6
C34—O8—H34	109.5	C27—C28—H28	120.6
N5—O9—Eu1	95.51 (15)	C30—C29—C28	122.8 (3)
N5—O10—Eu1	98.68 (14)	C30—C29—H29	118.6
C1—N1—C5	117.1 (2)	C28—C29—H29	118.6
C1—N1—Eu1	122.35 (16)	C29—C30—C31	119.2 (3)
C5—N1—Eu1	120.37 (15)	C29—C30—H30	120.4
C10—N2—C9	117.3 (2)	C31—C30—H30	120.4
C10—N2—Eu1	122.81 (17)	O3—C31—C30	117.3 (2)
C9—N2—Eu1	118.85 (15)	O3—C31—C26	122.5 (2)
C13—N3—C17	117.6 (2)	C30—C31—C26	120.2 (3)
C13—N3—Eu1	121.43 (17)	O6—C32—O5	119.7 (2)
C17—N3—Eu1	120.54 (15)	O6—C32—C33	121.0 (2)
C24—N4—C18	117.0 (2)	O5—C32—C33	119.3 (2)
C24—N4—Eu1	122.93 (16)	O6—C32—Eu1	61.87 (13)
C18—N4—Eu1	119.89 (16)	O5—C32—Eu1	57.83 (13)
O11—N5—O9	123.1 (3)	C33—C32—Eu1	176.62 (19)
O11—N5—O10	120.8 (2)	C34—C33—C38	117.7 (2)
O9—N5—O10	116.2 (2)	C34—C33—C32	121.0 (2)
O11—N5—Eu1	176.5 (2)	C38—C33—C32	121.3 (2)
O9—N5—Eu1	59.84 (12)	O8—C34—C35	117.9 (3)
O10—N5—Eu1	56.35 (12)	O8—C34—C33	121.2 (3)
N1—C1—C2	123.8 (2)	C35—C34—C33	120.9 (3)
N1—C1—H1	118.1	C36—C35—C34	119.6 (3)
C2—C1—H1	118.1	C36—C35—H35	120.2
C3—C2—C1	118.7 (3)	C34—C35—H35	120.2
C3—C2—H2	120.7	C35—C36—C37	121.8 (3)
C1—C2—H2	120.7	C35—C36—H36	119.1
C2—C3—C4	120.2 (2)	C37—C36—H36	119.1
C2—C3—H3	119.9	C36—C37—C38	119.8 (3)
C4—C3—H3	119.9	C36—C37—H37	120.1
C3—C4—C5	117.6 (2)	C38—C37—H37	120.1
C3—C4—C6	123.9 (2)	O7—C38—C37	117.9 (3)
C5—C4—C6	118.6 (3)	O7—C38—C33	122.0 (2)
N1—C5—C4	122.5 (2)	C37—C38—C33	120.2 (3)

### Hydrogen-bond geometry (Å, °)

**Table:**

*D*—H···*A*	*D*—H	H···*A*	*D*···*A*	*D*—H···*A*
O8—H34···O6	0.82	1.86	2.585 (3)	148
O4—H27···O2	0.82	1.83	2.561 (2)	148
O7—H38···O5	0.82	1.85	2.579 (3)	147
O3—H31···O1	0.82	1.87	2.594 (2)	147
